# Mutation in the RNA-Dependent RNA Polymerase of a Symbiotic Virus Is Associated With the Adaptability of the Viral Host

**DOI:** 10.3389/fmicb.2022.883436

**Published:** 2022-03-30

**Authors:** Hong Lu, Jing Li, Pengcheng Yang, Fei Jiang, Hongran Liu, Feng Cui

**Affiliations:** ^1^State Key Laboratory of Integrated Management of Pest Insects and Rodents, Institute of Zoology, Chinese Academy of Sciences, Beijing, China; ^2^CAS Center for Excellence in Biotic Interactions, University of Chinese Academy of Sciences, Beijing, China

**Keywords:** symbiotic virus, *Acyrthosiphon pisum* virus, RdRp, single nucleotide polymorphism, polymerase activity, pea aphid

## Abstract

Host adaptation has the potential to cause rapid genetic variation in symbiotic microorganisms in insects. How mutations in symbiotic viruses favor viral fitness in hosts and even influence host adaptability to new environments remains elusive. Here, we explored the role of genetic divergence at one site of a symbiotic virus, *Acyrthosiphon pisum* virus (APV), in the host aphid’s adaptation to unfavorable plants. Based on the transcriptomes of the pea aphid *Vicia faba* colony and *Vicia villosa* colony, 46 single nucleotide polymorphism (SNP) sites were found in the APV genomes from the two aphid colonies. One SNP at site 5,990, G5990A, located at the RNA-dependent RNA polymerase (RdRp) domain, demonstrated a predominance from G to A when the host aphids were shifted from *V. faba* to the low-fitness plants *V. villosa* or *Medicago sativa*. This SNP resulted in a substitution from serine (S) to asparagine (N) at site 196 in RdRp. Although S196N was predicted to be located at a random coil far away from conserved functional motifs, the polymerase activity of the N196 type of RdRp was increased by 44.5% compared to that of the S196 type. The promoted enzymatic activity of RdRp was associated with a higher replication level of APV, which was beneficial for aphids as APV suppressed plant’s resistance reactions toward aphids. The findings showed a novel case in which mutations selected in a symbiotic virus may confer a favor on the host as the host adapts to new environmental conditions.

## Introduction

RNA viruses are characterized by a high mutation rate due to the error-prone replication feature of the RNA-dependent RNA polymerase (RdRp; Elena and Sanjuan, 2005; Peck and Lauring, 2018). The mutation rate ranges from 10^−6^ to 10^−4^ substitutions per nucleotide per replication, and single-stranded RNA viruses mutate even faster (Duffy et al., 2008; Sanjuán et al., 2010). A high mutation rate and large viral population improves virus fitness for adapting to host conditions. The effects of mutations on viral pathogenicity in hosts have been frequently reported in some notorious viruses, such as the H7N9 avian influenza virus and SARS-CoV-2 (Huang and Wang, 2020; Awadasseid et al., 2021). Symbiotic viruses usually do not induce obvious diseases in hosts. How mutations in symbiotic viruses may favor viral fitness in hosts and even host adaptability to new environments remains elusive.

*Acyrthosiphon pisum* virus (APV) is a symbiotic virus of the pea aphid *A. pisum*, belonging to unclassified picorna-like single-stranded RNA viruses (van den Heuvel et al., 1997). The approximately 10 kb genome contains two open reading frames (ORFs). The ORF1 product contains domains of helicase, chymotrypsin-like protease, and RdRp. APV capsids (CPs) are composed of a 34 kD of major protein encoded by ORF1 and two minor proteins of 66 kD, encoded by ORF1 and ORF2 in a translational frameshift, and 23/24 kD most likely arising from the proteolytic breakdown of the 34 kD protein (Van den Heuvel et al., 1997). Our previous study demonstrated that APV facilitated pea aphids in adapting to unsuitable plants such as *Medicago truncatula* and *Vicia villosa* once the aphids were shifted from *Vicia faba* to these undesirable plants (Lu et al., 2016, 2020). One of the key mechanisms is that APV suppresses the titer of the phytohormone jasmonic acid after it is horizontally transferred to plants with aphid feeding (Lu et al., 2020). Furthermore, we found that the amount of APV in the pea aphid *M. truncatula* colony or the *V. villosa* colony was significantly higher than that in the *V. faba* colony (Lu et al., 2020). However, the mechanism for the variation in viral load in aphids during the adaptation process of aphids to their plants is not clear.

In this study, we retrieved the genome sequences of APV from previously sequenced salivary gland transcriptomes of the pea aphid *V. faba* colony and the *V. villosa* colony (Lu et al., 2016) and analyzed the single nucleotide polymorphism (SNP) sites in APV genomes. One SNP leading to a nonsynonymous mutation in RdRp demonstrated a significantly large ratio divergence between the two colonies. Enzymatic activity measurement of the two genotypes of RdRp showed that this mutation had a considerable effect on RNA polymerase activity and may account for the promoted viral replication level in aphids on unsuitable plants.

## Materials and Methods

### Aphids and Plants

The pea aphid *V. faba* colony, *V. villosa* colony, and APV-free *V. faba* colony were established as previously described (Wang et al., 2015; Lu et al., 2016, 2020) and raised in growth chambers at 21 ± 1°C, 60 ± 5% relative humidity, and a photoperiod of 16 h light:8 h dark. The field *M. sativa* colony was collected in Beijing in 2013.

### SNP Analysis of the APV Genome

Transcriptomic reads of the *V. faba* colony, *V. villosa* colony and *M. truncatula* colony (Lu et al., 2016) were aligned to the APV reference sequence (AF024514.1) by tophat2 (Kim et al., 2013). SNP sites were identified by “samtools mpileup” (v0.1.19; Li et al., 2009). Allele frequency was extracted from the vcf file, and the sequence logo was generated using WebLogo 3.^1^

### RNA Isolation and cDNA Synthesis

Total RNA was isolated from single aphids or five aphids using TRIzol Reagent (Invitrogen, Carlsbad, CA, United States) according to the manufacturers’ protocols. After treatment with the TURBO DNA-free kit (Ambion, Austin, TX, United States) to remove genomic DNA contamination, RNA was reverse-transcribed to cDNA using the SuperScript™ III first-strand synthesis system (Invitrogen) and Oligo-dT primers (Promega, Madison, Wisconsin, United States) in accordance with the manufacturers’ instructions.

### Reverse Transcription-PCR and Sanger Sequencing

RNA of 20 individual aphids from the *V. faba* colony, *V. villosa* colony, and the *V. villosa* colony that was raised on *V. faba* for 1–5 months and 27 individual aphids from the *M. sativa* colony were extracted for cDNA synthesis. A 460 bp fragment encompassing site 5,990 was amplified using the primer pair G5990A-F/G5990A-R (Supplementary Table S1) on a Mastercycler thermal cycler (Eppendorf, Hamburg, Germany). The PCR protocol was as follows: 95°C for 5 min; 35 cycles of 95°C for 30 s, 55°C for 30 s, and 72°C for 30 s; and 72°C for 5 min. The PCR products were sequenced by Sanger sequencing technology in Invitrogen (Beijing, China).

### RdRp Protein Sequence Alignment and *in silico* Structural Prediction

The amino acid sequences of APV RdRp were aligned with that of Kelp Fly virus RdRp using ClustalW at BioEdit.^2^ The three-dimensional structures of APV RdRp were predicted using Robetta^3^ and I-TASSER.^4^ In the Robetta server, RoseTTAFold was used for automated modeling. The confidence for the prediction was evaluated by the lDDT value, which represents accurate predictions when it is above 0.75 (Baek et al., 2021). In the I-TASSER server, five models were generated, and the best model was selected based on the threading sequence identity and C-score, which is typically in the range from −5 to 2. The predicted structures were visualized using PyMOL software (DeLano Scientific, San Carlos, CA, United States).

### RdRp Recombinant Protein Expression in *Escherichia Coli*

Two types of APV RdRp, S196, and N196, were subcloned into the pET28a vector to generate recombinant plasmids with His-tags using the primer pair RdRp-his-F/RdRp-his-R (Supplementary Table S1). The recombinant plasmids were transformed into *E. coli* BL21 (DE3) cells for protein expression. After overnight induction with 0.5 mM isopropyl β-D-thiogalactoside (IPTG) at 16°C, the cells were pelleted by centrifugation and resuspended in lysis buffer (20 mM Tris–HCl, pH 8.0) for sonication in ice water. The supernatant was retained for the RdRp enzymatic assay.

### RdRp Enzymatic Assay

A 101 bp fragment of APV *CP* containing a T7 RNA polymerase promoter at the 5′ terminus was amplified using cp101-T7F/cp101-R primers (Supplementary Table S1) and *in vitro* transcribed to *CP* RNA as an RNA template with T7 RNA polymerase (Promega) according to the manufacturer’s instructions. The enzymatic assay was performed in a total volume of 25 μl containing 20 mM Tris–HCl (pH 8.0), 1.5 mM MnCl_2_, 2 mM MgCl_2_, 100 mM NaCl, 4 mM DTT, 10 U RNasin (Promega), 250 μM NTP (including a biotin-labeled UTP; Roche, Basel, Switzerland), 0.5 μl of RNA template (800 ng/μl) and 100 μg of crude RdRp-His protein. The reaction mixture was incubated at 25°C for 2 h, and then 30 μg of proteinase K (Ambion) was added to terminate the reaction. The RNA products were extracted with phenol/chloroform (1:1), precipitated overnight using 95% ethanol containing 0.3 M sodium acetate and dissolved in 10 μl of RNase-free water.

The biotin-labeled RNA product was detected *via* dot blot. The RNA products were first spotted onto a BrightStar™-Plus positively charged nylon membrane (Thermo Scientific, Waltham, MA, United States) and then placed in a UV crosslinker (Spectronics Corporation, Westbury, NY, United States) for 10 min. The biotin-labeled RNA products were detected with a Chemiluminescent Nucleic Acid Detection Module Kit (Thermo Scientific) according to the manufacturer’s instructions. The RdRp-His protein in the crude extracts was measured by western blotting using an anti-His monoclonal antibody (CWBiotech, Beijing, China). The RdRp enzyme activity was represented by the relative intensity of biotin-labeled RNA products to RdRp-His, which was quantified with ImageJ software. The 1 mM and 0.1 mM biotin-16-UTP were used as positive controls. One hundred micrograms of crude protein from the cells transformed with pET28a vector and 20 mM Tris–HCl (pH 8.0) buffer were used as negative controls. Six replicates were prepared. Differences were analyzed using Student’s *t* test with SPSS 17.0 software.

### Quantification of APV Load by Real-Time Quantitative PCR

APV load in the following three groups of aphids was measured using qPCR: the third-instar nymphs of the *V. faba* colony and *V. villosa* colony; the APV-free third-instar nymphs that were fed on an artificial diet containing the APV crude preparations from the *V. faba* colony or *V. villosa* colony for 1 d (Lu et al., 2020) and then raised on *V. villosa* for 8 d; and the third-instar nymphs of the *V. villosa* colony that were raised on *V. faba* for 3, 4, and 5 months. Five to nine biological replicates with five aphids per replicate or from 13 to 19 individual aphids were prepared for RNA extraction. qPCR was applied to quantify the RNA level of APV *CP* with the primer pair cp-F/cp-R (Supplementary Table S1) on a Light Cycler 480 II instrument (Roche). The transcript level of ribosomal protein L27 (NM_001126221) was quantified with primer pair L27-F/L27-R (Supplementary Table S1) as an internal control. The thermal cycling conditions were 95°C for 2 min, 40 cycles of 95°C for 20 s, 55°C for 20 s, and 68°C for 20 s, followed by one cycle of 95°C for 30 s, 58°C for 30 s, and 95°C for 10 s to determine the melting curve. The relative RNA level of APV *CP* to the transcript level of *L27* is represented as the mean ± SE. Differences were analyzed using Student’s *t* test for pairwise comparisons with SPSS 17.0 software.

## Results

### Single Nucleotide Polymorphism Sites in APV Genomes From Two Pea Aphid Colonies

**Figure 1:**
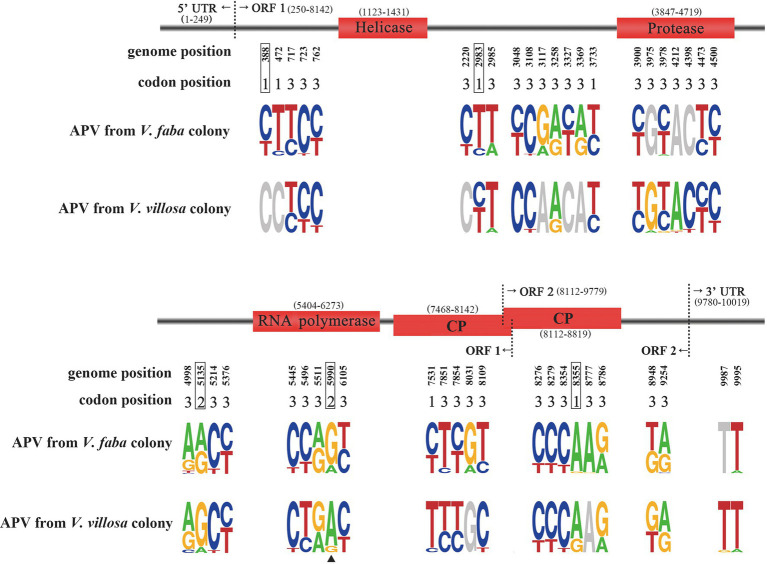
Sequence logo showing allele frequencies at 46 SNP sites in APV genomes from the pea aphids *Vicia faba* colony and *Vicia villosa* colony. The genome position and corresponding codon position for each SNP are shown. Four domains are marked in red rectangles. Five SNPs resulting in nonsynonymous mutations are boxed. Site 5,990 is marked with a triangle. A logo represents each column of the alignment by a stack of letters, with the height of each letter proportional to the observed frequency of the corresponding nucleotide.

We assembled the APV genomes from the previously sequenced salivary gland transcriptomes of the pea aphid *V. faba* colony and *V. villosa* colony (Lu et al., 2016). The assembled APV genome (registration number of OM812681 in GenBank) had 10,019 nucleotides (nt), including a 249 nt 5′-untranslated sequence and a 240 nt 3′-untranslated sequence containing a polyadenine. Following alignment of the reference APV genome (van der Wilk et al., 1997), four domains were identified, i.e., helicase from 1,123 to 1,431 nt, chymotrypsin-like protease from 3,847 to 4,719 nt, RdRp from 5,404 to 6,273 nt, and CP from 7,468 to 8,819 nt (Figure 1).

A total of 46 SNP sites were found in the APV genomes from the *V. faba* colony and *V. villosa* colony (Figure 1; Supplementary Table S2). Five SNPs at nucleotides 388, 2,983, 5,135, 5,990, and 8,355 were supposed to change amino acids, and the remaining 41 SNPs were synonymous mutations. Among the five nonsynonymous SNPs, site 5,990 was located in the RdRp domain, and site 8,355 was in the CP domain. Furthermore, the predominant nucleotides at site 5,990 were significantly different between the APV genomes from the *V. faba* colony and the *V. villosa* colony, i.e., 84% of G in the *V. faba* colony and 87% of A in the *V. villosa* colony, leading to a change in amino acid residues from serine (S) to asparagine (N). In the APV genome from the *M. truncatula* colony, which was previously sequenced (Lu et al., 2016), the nucleotides at site 5,990 were 62% of G and 38% of A. On the other hand, the predominant nucleotides at site 8,355 were the same in the two colonies, i.e., 96% of A in the *V. faba* colony and 73% of A in the *V. villosa* colony. For the other three nonsynonymous SNPs at sites 388, 2,983, and 5,135, the ratios of predominant nucleotides were not as divergent as site 5,990 between the two colonies.

### Divergence of SNP at Site 5,990 of APV in Different Colonies of Pea Aphids

**Figure 2:**
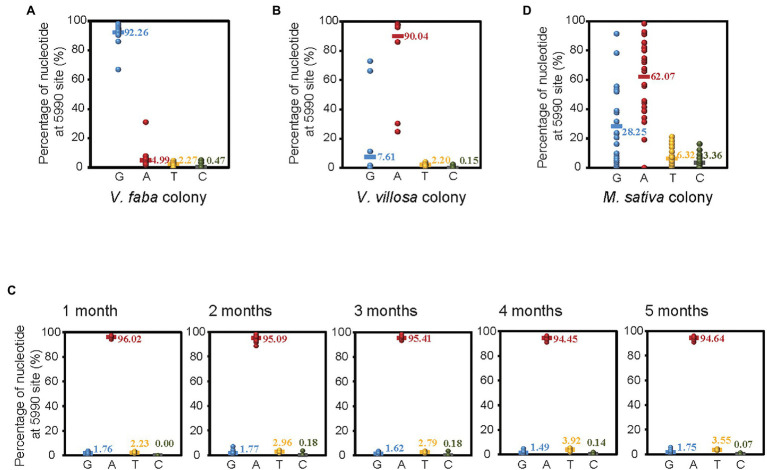
Divergence of SNP at site 5,990 of APV in different colonies of pea aphids. **(A)** *Vicia faba* colony. **(B)** *Vicia villosa* colony. **(C)** *V. villosa* colony that was raised on *V. faba* for 1–5 months. **(D)** *Medicago sativa* colony. Twenty individual pea aphids were sequenced in **(A–C)**, and 27 individuals were sequenced in **(D)**.

To verify the different ratios of G or A at site 5,990 of APV in different colonies of pea aphids, we amplified and sequenced a 460 bp fragment encompassing site 5,990 with RT–PCR and Sanger sequencing in 20 individual pea aphids from both the *V. faba* colony and the *V. villosa* colony, which was acclimated 14 months after shifting from *V. faba*. In the *V. faba* colony, the ratio of G at site 5,990 was 92.26%, and the ratio of A was 4.99% (Figure 2A), while in the *V. villosa* colony, the ratio of G was 7.61%, and the ratio of A was 90.04% (Figure 2B). This result was consistent with that from the transcriptomes, suggesting that the predominant nucleotide at site 5,990 of APV was changed from G to A when the *V. faba* colony was shifted and adapted to the unsuitable *V. villosa*. However, when the *V. villosa* colony was transferred back to *V. faba* and raised for 5 months, the predominant nucleotide at site 5,990 of APV was still A, with a ratio no lower than 94% in 20 aphids sampled every month (Figure 2C). Furthermore, we sequenced the 460 bp fragment of APV from a field colony of pea aphids living on *Medicago sativa*, which is a low-fitness plant for pea aphids (Lu et al., 2016). In the 27 individuals, 62.07% of the nucleotides at site 5,990 of APV were A, and 28.25% were G (Figure 2D). Therefore, an obvious divergence in the predominant nucleotides at site 5,990 of APV occurred when the pea aphids adapted to different plants.

### Location of Site 5,990 in the RdRp Structure

**Figure 3:**
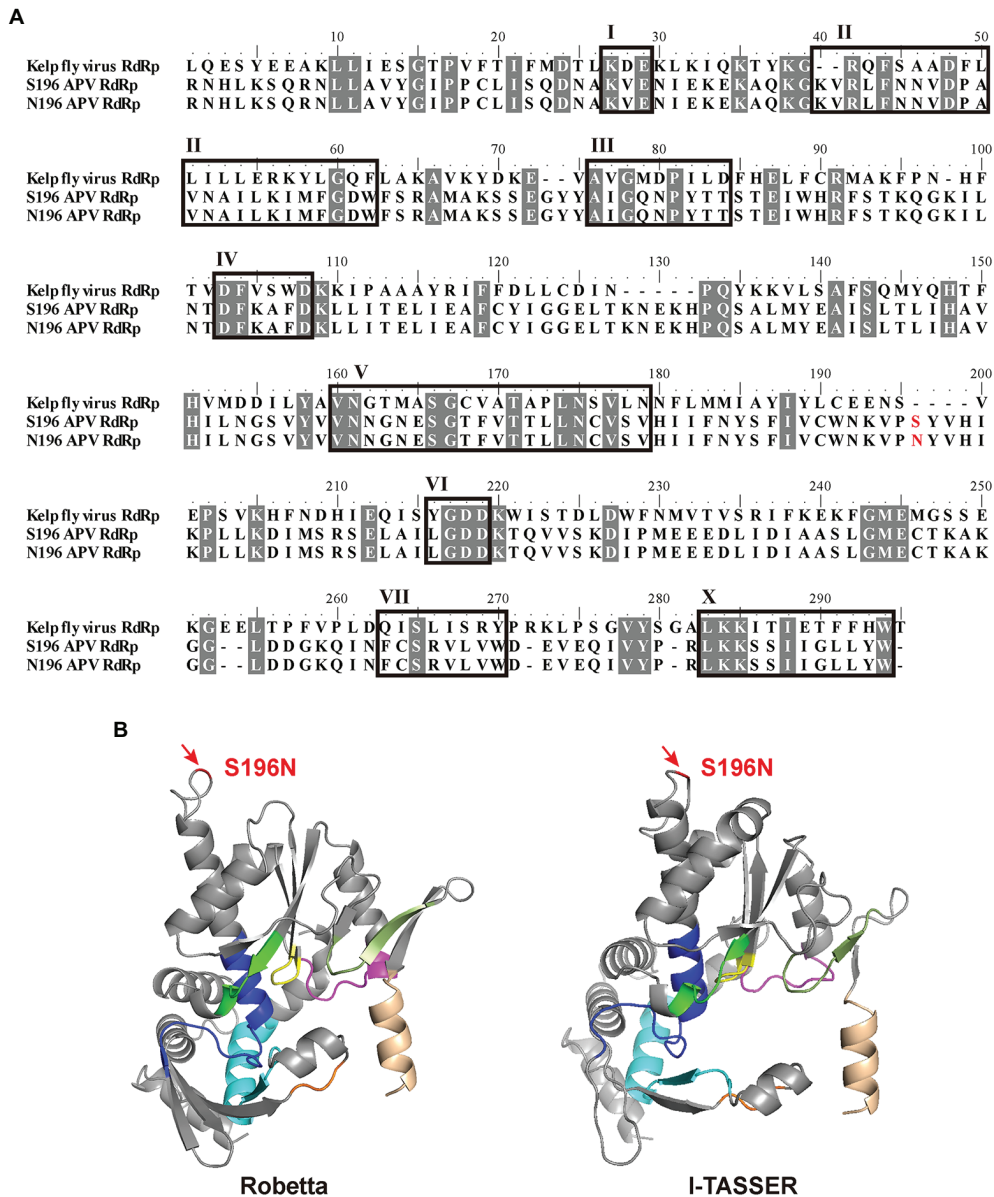
Location of site 5,990 in the structure of RdRp. **(A)** Alignment of amino acid sequences among two types of APV RdRp and Kelp fly virus RdRp. Eight conserved motifs (I–VII, X) are boxed. The S or N at amino acid residue 196 is shown in red. Identical or similar amino acid residues among the three sequences are shaded in gray. **(B)** Three-dimensional structures of APV RdRp predicted *in silico* by the Robetta and I-TASSER servers. Motifs are shown in assorted colors: I, orange; II, cyan; III, magenta; IV, green; V, blue; VI, yellow; VII, smudge; X, wheat. Site S196N is marked in red.

With alignment to the RdRp amino acid sequence of Kelp fly virus (YP_415507), which is also a picorna-like virus (Hartley et al., 2005), APV RdRp contained eight conserved motifs (I–VII, X; Figure 3A). The D-X (4 or 5)-D in motif IV and the GDD in motif VI bind the divalent cations Mg^2+^ and/or Mn^2+^, and GDD also serve as crucial catalytic residues for RNA polymerase activity (Ng et al., 2002). Motif V determines the synthesis of RNA or DNA by discriminating NTPs from dNTPs (Gohara et al., 2000). The S or N at amino acid residue 196 of RdRp corresponding to genomic nucleotide site 5,990 was between motifs V and VI in the primary structure (Figure 3A). The three-dimensional structures of APV RdRp predicted *in silico* by the Robetta and I-TASSER servers were similar (Figure 3B), although the confidence levels were marginally acceptable, with a local distance difference test (lDDT) value of 0.78 in the Robetta prediction and a C-score of 0.65 in the I-TASSER prediction. Site S196N was located at a random coil far away from the eight conserved motifs (Figure 3B).

### Effects of SNP at Site 5,990 on the Enzymatic Activity of APV RdRp

**Figure 4:**
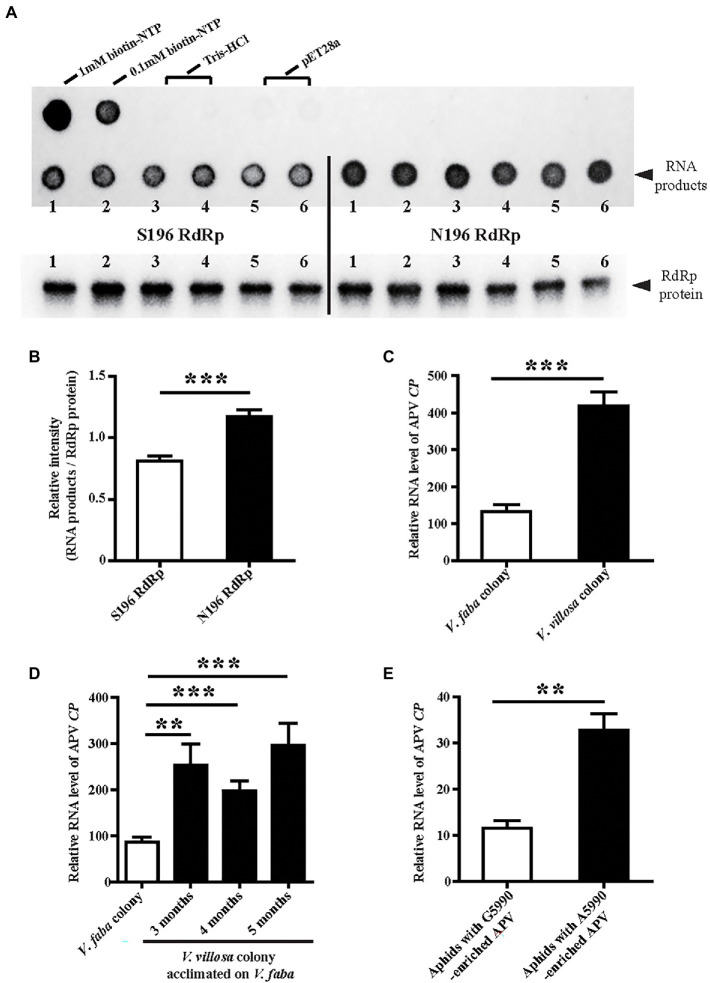
Effects of SNP at site 5,990 on the enzymatic activity of APV RdRp and viral replication levels. **(A)** Dot blots showing the biotin-labeled RNA products synthesized by the recombinantly expressed S196-type RdRp-His and N196-type RdRp-His. The 1 and 0.1 mM biotin-NTP were used as positive controls. One hundred micrograms of crude protein from the cells transformed with pET28a vector and 20 mM Tris–HCl (pH 8.0) buffer were used as negative controls. The RdRp-His proteins used in the assays were measured by western blotting with an anti-His monoclonal antibody. Six replicates were prepared. **(B)** The relative intensity of synthesized RNA products to that of RdRp-His from **(A)** quantified with ImageJ software. The values represent the means ± SEs. **(C)** Comparison of the relative RNA levels of APV *CP* between the *V. faba* colony and *V. villosa* colony. Five to six biological replicates were prepared. **(D)** Comparison of the relative RNA levels of APV *CP* in the *V. villosa* colony that was acclimated on *V. faba* for 3–5 months. Thirteen to nineteen individual aphids were measured. **(E)** Comparison of the relative RNA levels of APV *CP* in the aphids 8 days post-infection with the A5990-enriched APV and the G5990-enriched APV. Nine biological replicates were prepared. The relative RNA level of APV *CP* to the transcript level of *L27* is represented as the mean ± SE. Differences were analyzed using Student’s *t* test. **p < 0.01; ***p < 0.001.

To assess whether the substitution S196N at nucleotide site 5,990 affects enzymatic activity, two types of APV RdRp, S196 and N196, were recombinantly expressed in *E. coli* cells and used for enzymatic activity measurement with an RNA template and biotin-labeled NTP. The amounts of synthesized RNA products were compared between the two types of RdRp after normalization by the RdRp protein amounts. The results showed that the N196 type of RdRp produced more RNA products than the S196 type (Figure 4A), indicating that the substitution of G by A at site 5,990 increased the enzymatic activity of APV RdRp by 44.5% (Figure 4B).

### Effects of SNP at Site 5,990 on Viral Replication Levels

The APV loads with different genotypes of RdRp were further explored in different aphid colonies using qPCR. The amount of APV in the *V. villosa* colony was approximately 3-fold that in the *V. faba* colony in terms of the RNA level of APV *CP* after 14 months of acclimation on *V. villosa* (Figure 4C). Even though the *V. villosa* colony was transferred back to *V. faba* and raised for 3–5 months, the predominant nucleotide at site 5,990 was still A (Figure 2C), and the amount of APV was significantly higher than that in the *V. faba* colony (Figure 4D). When the APV-free aphids were fed an artificial diet containing A5990-enriched APV from the *V. villosa* colony or G5990-enriched APV from the *V. faba* colony, a remarkably higher viral load was detected in the aphids 8 d post-infection with the A5990-enriched APV than that following infection with the G5990-enriched APV (Figure 4E). Considering the fact that the substitution of Ser by Asn (S196N) increased the enzymatic activity of RdRp, the higher replication level of the A5990-enriched APV could result from the promoted enzymatic activity of RdRp.

## Discussion

In this study, we found that a mutation in the RdRp of the symbiotic virus APV promoted polymerase activity, leading to an increased viral replication level in the host aphids. This mutation was preferred when aphids colonized unsuitable plants. The findings showed a novel case in which mutations selected in a symbiotic virus may confer a favor on the host as the host adapts to new environmental conditions.

The driving force for the S196N substitution in APV RdRp originated from the adaptation ability of host aphids to their plants. The S196N substitution enhanced APV replication in aphids so that more viruses were secreted to plants, which suppressed jasmonic acid-mediated resistance to aphids (Lu et al., 2020). This suggests that the symbiotic APV was in a similarly stressed situation as its host aphid during the aphid’s adaptation to an unfamiliar environment, and the selection pressure on RdRp genotypes originated from the plants of host aphids. This case is different from the established theory of mutations in viral polymerases and surface glycoproteins, which are regarded as favorable for viral adaptability to new hosts. For example, nearly 80% of H5N1 influenza A virus isolates contain substitutions E627K, K526R, or D701N in the PB2 polymerase, leading to the enhancement of polymerase activity, virus replication and cross-species transmission (Hatta et al., 2001; Song et al., 2014). Similarly, the substitution D614G in the spike protein of SARS-CoV-2 increased viral transmission by 43–90% (Winger and Caspari, 2021), and the substitutions A226V in the envelope glycoprotein E1 and L210Q in E2 increased the infectivity of chikungunya virus in *Aedes albopictus* (Tsetsarkin et al., 2007; Tsetsarkin and Weaver, 2011).

The substitution at the S196N site had a profound influence on the enzymatic activity of APV RdRp. Viral RdRp usually contains palm, thumb and finger subdomains (Hansen et al., 1997). Motifs IV, V, and VI are localized in the palm subdomain and show unequivocal conservation throughout positive-stand RNA viruses (Poch et al., 1989; Koonin and Dolja, 1993). *In vitro* mutations within the palm subdomain usually dramatically reduce or eliminate RdRp activity in viruses, such as encephalomyocarditis virus (Sankar and Porter, 1992), hepatitis C virus (Lohmann et al., 1997), Zika virus and Dengue virus (Xu et al., 2017). Alternatively, the substitution R345K enhances the RdRp activity of hepatitis C virus by approximately 50% (Lohmann et al., 1997). The S196N site is between motifs V and VI in the primary structure of APV RdRp but far away from the palm region viewed from the simulated three-dimensional structure. The inconsequential spatial location seems inadequate to explain the enhancement of RdRp activity with the S196N substitution unless the simulation was not sufficiently accurate considering the low conservation of the sequences in the region around the S196N site. Resolution of the genuine three-dimensional structure of APV RdRp would be enormously helpful in solving this puzzle.

## Conclusion

The symbiotic virus APV changed as its host aphid acclimated to an unfavorable plant. The aphid’s adaptation on unfavorable plants appears to have enhanced the selection of a mutation in viral RdRp that promoted APV replication, which may facilitate the aphid’s adaptation process by suppressing the plant’s resistance reaction toward aphids, representing a new evolutionary model of viruses in nature.

## Data Availability Statement

The original contributions presented in the study are included in the article/Supplementary Material, and further inquiries can be directed to the corresponding author.

## Author Contributions

HL did most experiments, wrote original draft, and predicted RdRp structure. JL and PY analyzed SNPs. FJ helped to measure RdRp activity. FC conceptualized and supervised the study and finalized the paper. All authors contributed to the article and approved the submitted version.

## Funding

This work was supported by grants from the Strategic Priority Research Program of Chinese Academy of Sciences (no. XDA26050404) and the National Natural Science Foundation of China (no. 32102207).

## Conflict of Interest

The authors declare that the research was conducted in the absence of any commercial or financial relationships that could be construed as a potential conflict of interest.

## Publisher’s Note

All claims expressed in this article are solely those of the authors and do not necessarily represent those of their affiliated organizations, or those of the publisher, the editors and the reviewers. Any product that may be evaluated in this article, or claim that may be made by its manufacturer, is not guaranteed or endorsed by the publisher.

## Footnotes

^1^ http://weblogo.threeplusone.com

^2^ http://www.mbio.ncsu.edu/BioEdit/

^3^ https://robetta.bakerlab.org/

^4^ https://zhanggroup.org/I-TASSER/
