# Supplementary material for: Mutation in the RNA-Dependent RNA Polymerase of a Symbiotic Virus Is Associated With the Adaptability of the Viral Host
Source: Front Microbiol. 2022 Mar 30;13:883436. doi: 10.3389/fmicb.2022.883436 (PMC9005967; doi:10.3389/fmicb.2022.883436)
Supplement: Supplementary file 1 [file Table_1.DOCX]

Table S1. Primer sequences used in this study

| Primer name | Primer sequence (5’ to 3’) | Product length (bp) |
| --- | --- | --- |
| G5990A-F | CCTTACACAACATCCACAGAGAT | 460 |
| G5990A-R | CTTCTTCTTCCATCGGTATGTCTT |  |
| RdRp-his-F | AATGGGTCGCGGATCCATGAGAAACCATCTTAAATCAC | 905 |
| RdRp-his-R | GGTGGTGGTGCTCGAGCCAATATAGTAAGCCAATAATAC |  |
| cp101-T7F | TAATACGACTCACTATAGGTTCATCAGAGTGGAGTCATAC | 101 |
| cp101-R | CATCGTCTACTTGAGTCTTCT |  |
| cp-F | TCGGAGACAACAGTACCTATGG | 168 |
| cp-R | GCTTCCTACATCGTCTACTTGAG |  |
| L27-F | TCGTTACCCTCGGAAAGTC | 108 |
| L27-R | GTTGGCATAAGGTGGTTGT |  |
