# Supplementary material for: Mutation in the RNA-Dependent RNA Polymerase of a Symbiotic Virus Is Associated With the Adaptability of the Viral Host
Source: Front Microbiol. 2022 Mar 30;13:883436. doi: 10.3389/fmicb.2022.883436 (PMC9005967; doi:10.3389/fmicb.2022.883436)
Supplement: Supplementary file 2 [file Table_2.DOCX]

Table 1. The genetic divergence of APV genome sequences in *Vicia faba* colony and *Vicia villosa* colony. All 46 single nucleotide polymorphism (SNP) sites were listed in genomic order.

|  | *Vicia faba* colony | | | | *Vicia villosa* colony | | | |
| --- | --- | --- | --- | --- | --- | --- | --- | --- |
| Genome position | A | T | G | C | A | T | G | C |
| 388 |  | 29% |  | 71% |  |  |  | 100% |
| 472 |  | 90% |  | 10% |  |  |  | 100% |
| 717 |  | 67% |  | 33% |  | 60% |  | 40% |
| 723 |  | 5% |  | 95% |  | 27% |  | 73% |
| 762 |  | 35% |  | 65% |  | 15% |  | 85% |
| 2220 |  | 23% |  | 77% |  |  |  | 100% |
| 2983 |  | 85% |  | 15% |  | 50% |  | 50% |
| 2985 | 25% | 75% |  |  | 10% | 90% |  |  |
| 3048 |  | 45% |  | 55% |  |  |  | 100% |
| 3108 |  | 4% |  | 96% |  | 8%% |  | 92% |
| 3117 | 20% |  | 80% |  | 100% |  |  |  |
| 3258 | 67% |  | 33% |  | 73% |  | 27% |  |
| 3327 |  | 50% |  | 50% |  |  |  | 100% |
| 3369 | 64% |  | 36% |  | 100% |  |  |  |
| 3733 |  | 58% |  | 42% |  | 64% |  | 36% |
| 3900 |  | 46% |  | 54% |  | 56% |  | 44% |
| 3975 |  |  | 100% |  | 5% |  | 95% |  |
| 3978 | 3% | 45% |  | 52% |  | 56% | 4% | 40% |
| 4212 | 100% |  |  |  | 94% | 3% | 3% |  |
| 4398 |  |  |  | 100% |  | 8% |  | 92% |
| 4473 |  | 67% |  | 33% |  | 50% |  | 50% |
| 4500 |  | 37% |  | 63% |  | 24% |  | 76% |
| 5088 | 72% | 5% | 21% | 2% | 57% |  | 36% | 7% |
| 5135 | 55% |  | 45% |  | 12% |  | 88% |  |
| 5214 |  | 11% |  | 89% |  | 6% |  | 94% |
| 5376 |  | 45% |  | 55% |  | 50% |  | 50% |
| 5445 |  | 12% |  | 88% |  | 9% |  | 91% |
| 5496 |  | 23% |  | 77% |  | 70% |  | 30% |
| 5511 | 50% |  | 50% |  | 40% |  | 60% |  |
| 5990 | 16% |  | 84% |  | 87% |  | 13% |  |
| 6105 |  | 52% |  | 48% |  | 31% |  | 69% |
| 7531 |  | 36% |  | 64% |  | 91% |  | 9% |
| 7851 |  | 89% |  | 11% |  | 58% |  | 42% |
| 7854 |  |  | 50% | 50% |  | 57% |  | 43% |
| 8031 | 16% |  | 84% |  |  |  | 100% |  |
| 8109 |  | 76% |  | 24% |  | 10% |  | 90% |
| 8276 |  | 20% |  | 80% |  | 24% |  | 76% |
| 8279 |  | 20% |  | 80% |  | 26% |  | 74% |
| 8354 |  | 17% |  | 83% |  | 9% |  | 91% |
| 8355 | 96% |  | 4% |  | 73% |  | 27% |  |
| 8777 | 98% |  | 2% |  | 100% |  |  |  |
| 8786 | 39% |  | 61% |  | 30% |  | 70% |  |
| 8948 |  | 56% | 44% |  |  | 41% | 59% |  |
| 9254 | 62% |  | 36% | 2% | 60% |  | 40% |  |
| 9987 |  | 100% |  |  |  | 95% | 5% |  |
| 9995 | 6% | 94% |  |  | 7% | 93% |  |  |
